# Value of the stroke 1-2-0 prehospital stroke education system: the experience of a general practitioner team

**DOI:** 10.1186/s12883-023-03476-0

**Published:** 2023-12-07

**Authors:** Yang Liu, Daosheng Wang, Min Chu, Zhenzhen Yang, Yunhe Luo, Delong Wang, Jing Zhao

**Affiliations:** 1 Department of Neurology, Minhang Hospital, Fudan University, Shanghai, 201100 China; 2 Institute of Science and Technology for Brain-inspired Intelligence, Fudan University, Shanghai, China; 3 Department of Neurosurgery, Minhang Hospital, Fudan University, Shanghai, China; 4 Department of General Medicine, Xinzhuang Community Health Service Center, Shanghai, China

**Keywords:** Stroke 1-2-0, General practitioner, Prehospital training, Ischemic Stroke

## Abstract

**Background:**

Stroke is one of the leading causes of death worldwide, especially in developing countries. In China, there is an urgent need to educate people about stroke awareness and the importance of using emergency medical services (EMS) quickly after a stroke has occurred.

**Objective:**

We sought to explore the effects of the Stroke 1-2-0 Prehospital Stroke Education System based on the experience of a general practitioner team.

**Method:**

We prospectively enrolled 119 community general practitioners to be trained in the procedures advocated by the Stroke 1-2-0 Prehospital Stroke Education System. The training content included early detection of ischemic stroke, first aid for stroke, and intravenous thrombolysis; The effects of the training were later evaluated via a before-and-after comparison. The 119 enrolled physicians formed a Stroke 1-2-0 lecturer group and taught stroke knowledge to community residents. The group remained active for 6 months, during which the medical treatment data of stroke patients (i.e., stroke onset time, prehospital delay, whether an ambulance was called, and whether thrombolytic therapy was performed) in each of 5 jurisdictions were recorded for the month before (January 2021) and that after (August 2021) the 6-month community education program. Finally, the effects of the community education program were evaluated.

**Results:**

The participants’ understanding of intravenous thrombolysis in the treatment of acute ischemic stroke improved significantly after the training as compared with their earlier understanding (96% vs. 78.99%; *P* < .001), and their understanding of the time window for intravenous thrombolysis increased from 26.05% before to 72% (*P* < .001) after the training. Most of the participants (90% vs. 67.23%; *P* < .001) said that they would immediately call the 120 emergency number of China’s emergency phone system if they encountered individuals who appeared to be victims of acute stroke. A total of 82 stroke patients were seen before and 67 after the community education program. As for the use of the emergency call system, more patients with stroke activated that system after the program versus before (21.95% vs. 37.31%; *P* = .04). The 3-hour arrival rate after the program was nearly three times higher than that before the program (62.69% vs. 19.51%; *P* < .001). Also, regarding receiving thrombolysis after the occurrence of a stroke, the program triggered a substantial increase compared with the total earlier (19.4% vs. 6.1%; *P* = .013).

**Conclusion:**

We found that the Stroke 1-2-0 Prehospital Stroke Education System significantly improved community residents’ knowledge regarding stroke.

## Introduction

The main cause of death and disability in China is cerebral infarction, which is brought on by an immediate blockage of cerebral blood arteries and rapidly reduces the blood flow to specific brain tissue. The latest published epidemiologic data show that 1 in every 5 deaths in China is due to cerebrovascular disease. The current incidence and prevalence of stroke in China have reached 276.7 and 2022.0 per 100,000 people, respectively [1]. With the rapid aging of China’s population, the burden of disease due to stroke will continue to increase. The key to controlling this number is for patients to seek medical treatment in a timely manner and to obtain the most effective treatment as promptly as possible. The question of how to implement these goals has always been the focus of research around the world. Other countries have long utilized.

the FAST (Face, Arm, Speech, Time) identification method [2], which has been highly praised. In the past, European and American countries have used FAST for stroke education and achieved good results [3–7]. However, when FAST was translated into Chinese for use in China, it was not effective [8]. Therefore, in 2016, Our team first proposed the Stroke 1-2-0 early identification rule for stroke. 1 means to first check whether the corners of the mouth are skewed, 2 to check whether the unilateral limbs are weak, and 0 to listen to whether the person’s speech is slurred. If these symptoms appear, the practitioner must immediately call the 120 emergency number. This protocol was published in the previous study [9], filling in the gap for the early identification of stroke in China. The 1-2-0 principle facilitates the localization of stroke education by associating the initial symptoms of the condition with the emergency system’s telephone number 120 in China. We anticipate its imminent acceptance among the people of China.

General practitioners, as gatekeepers of the health of their communities, play an important role in the health care of local residents. Previous research has demonstrated that stroke education for these physicians can improve the rehabilitation of poststroke patients while also raising community knowledge of the prevalent risk factors for stroke [10–12]. However, there is no research on whether Stroke 1-2-0 can easily be integrated into the general practitioner’s stroke education system. Therefore, the objective of this study was to explore the application of the Stroke 1-2-0 education system to community general practitioners in China and its impact on raising stroke awareness and improving the use of emergency medical services among community residents.

## Methods

The study protocol was approved by the ethics committee of the Central Hospital of Minhang District, Shanghai, China.

### General information

In January 2021, we invited 120 general practitioners from 5 communities working in the suburbs of Minhang district to participate in training to be part of the Stroke 1-2-0 prehospital education team. The requirement for general practitioners included in the training was to complete general practitioners’ standardized training for resident physicians or to have had 1 or more years of community work. The training lasted 1 day and was conducted offline. The participants completed a stroke knowledge questionnaire online both before and after the training to evaluate its effects.

After the formation of the Stroke 1-2-0 lecturer group, from February 2021 to July 2021, we started the half-year Stroke 1-2-0 stroke community education program. The general practitioner team gave lectures in communities and outpatient clinics once a month, and put up posters about stroke knowledge on community bulletin boards. They checked the community cardiovascular and cerebrovascular disease review system and collected the baseline characteristics and medical treatment data of stroke patients in each jurisdiction for the month before the education program (January 2021) and the month after the education program (August 2021). Before and after the educational activity, data from 82 to 67 stroke patients were included in the evaluation. These data included the time of stroke onset, when the hospital was visited, whether an ambulance was called, and whether thrombolytic therapy was given.

### Data collection

The stroke knowledge questionnaire used in the training of community physicians was designed with the Questionnaire star system to record baseline characteristics, such as age, gender, and education, and to examine the physicians’ understanding of intravenous thrombolytic therapy and emergency measures after stroke, as well as the meaning of Stroke 1-2-0. To acquire the data of stroke patients before and after the community education program, data after the program were prospectively collected and the data before the program were gathered from the medical records and interviewing the patients and their families. Specifically, we first checked the stroke-verification system to collect data about the patients who had suffered a stroke in January 2021, and then the general practitioner team used a door-to-door or outpatient verification method to check each patient’s discharge information. The onset and arrival times were estimated by the patients and their family members. They were also asked about whether or not they had called the emergency phone number. General information and past illness information were verified in the discharge summary, which also stated whether or not thrombolytic therapy was received.

### Statistical method

SPSS version 25.0 (IBM Corporation, Armonk, NY) was used to analyze the data. Categorical variables were described as frequency and percentage and the statistical method was the chi-squared test or Fisher’s exact test. Continuous variables were described as the mean ± standard deviation (SD), and the data with normal distribution and homogeneity of variance underwent a *t* -test for analysis. Statistical significance was set at a two-tailed *P* value of < 0.05.

## Results

### Stroke 1-2-0 general practitioner education team

A total of 120 community general practitioners were invited and 119 (99.17%) agreed to participate in stroke knowledge training. Table 1 presents their baseline characteristics. Their average age was 36 years, 45.38% had intermediate professional titles, and 57.14% had at least a bachelor’s degree. After the training, 100 of the participants responded to our questionnaire (83.33%). There was no significant difference in age, gender, professional title, or educational background between the participants who responded before and after the training (*P* > .05).

**Table 1:** Characteristics of the community general practitioners who participated in the Stroke 1-2-0 education training

		Before education	After education		
		119	100	*P*	
Age		36.33 ± 6.06	36.78 ± 5.98	0.581	
Gender	Male	41 (34.45%)	35 (35.00%)	0.933	
	Female	78 (65.55%)	65 (65.00%)		
Job title	Entry level	43 (36.13%)	39 (39.00%)	0.907	
	Mid-level	54 (45.38%)	43 (43.00%)		
	High level	22 (18.49%)	18 (18.00%)		
Education	Technical secondary school	21 (17.65%)	18 (18.00%)	0.930	
	Junior college	30 (25.21%)	24(24.00%)		
	College and above	68 (57.14%)	58 (58.00%)		

Table 2 presents the impact of training on the stroke-related knowledge of community physicians. Regarding the understanding of intravenous thrombolysis in the treatment of acute ischemic stroke, the knowledge level post-training was significantly higher compared with that before the training (96% vs. 78.99%; *P* < .001). Also, the understanding of the time window of intravenous thrombolysis increased from 26.05% before the training to 72% after the training (*P* < .001). After training, More community physicians said that they would immediately call the 120 emergency number, thus shortening the prehospital delay, when they encountered individuals who appeared to be experiencing a stroke (90% vs. 67.23%; *P* < .001). Regarding the understanding of the Stroke 1-2-0 stroke education tool, almost every responding community physician could answer the questions contained in the Stroke 1-2-0 educational protocol after the training. This has paved the way for community residents’ stroke education.

**Table 2:** Changes in knowledge of stroke among community physicians who participated in the Stroke 1-2-0 training

	Answer	Before education	After education	*P*
		119	100	
Do you know the status of intravenous thrombolysis in stroke treatment?	I don’t understand	25 (21.01%)	4 (4.00%)	< 0.001
	I understand	94 (78.99%)	96 (96.00%)	
Do you know the time window for intravenous thrombolysis in ischemic stroke?	I don’t know	88 (73.95%)	28 (28.00%)	< 0.001
	I know	31 (26.05%)	72 (72.00%)	
If someone around you had a stroke, would you call an ambulance immediately?	I wouldn’t	39 (32.77%)	10 (10.00%)	< 0.001
	I would	80 (67.23%)	90 (90.00%)	
Do you know the specifics of “Stroke 1-2-0” for quick stroke identification?	I can say it all	52 (43.70%)	99 (99.00%)	< 0.001
	I can say some of it	32 (26.89%)	1 (1.00%)	
	I can’t say any of it	35 (29.41%)	0	

### Evaluation of the effect on community residents before and after receiving stroke education

We compared the data on stroke patients the month before and again the month after the Stroke 1-2-0 community education program conducted by the general practitioners in their respective communities. Table 3 presents the baseline data of patients before and after the program. There were 82 stroke patients before the program and 67 stroke patients after the program. There was no significant difference in the baseline characteristics of the stroke patients before and after the program (*P >* .05).

**Table 3:** Baseline characteristics of stroke patients before and after the education program

		the month before education	the month after education	
	N	82	67	*P*
Age		69.36 ± 11.22	66.67 ± 11.96	0.159
Gender	Male	57 (69.51%)	55 (82.09%)	0.077
	Female	25 (30.49%)	12 (17.91%)	
Alcoholic	Yes	8 (9.80%)	5 (7.46%)	0.622
	No	74(90.20%)	62 (92.54%)	
Smoking	Never smoked	64 (78.05%)	44 (65.67%)	0.107
	Previous smoker but not now	0 (0%)	2 (2.99%)	
	Always smoked	18 (21.95%)	21 (31.34%)	
Medical history				
Cerebral infarction	Yes	14 (17.07%)	14 (20.90%)	0.552
	No	68 (82.93%)	53 (79.10%)	
Hypertension	Yes	60 (73.17%)	45 (67.16%)	0.424
	No	22 (26.83%)	22 (32.84%)	
Diabetes	Yes	30 (36.59%)	18 (26.87%)	0.207
	No	52 (63.41%)	49 (73.13%)	
Atrial fibrillation	Yes	5 (6.10%)	6 (8.96%)	0.507
	No	77 (93.90%)	61 (91.04%)	
Hyperlipidemia	Yes	9 (10.98%)	10 (14.93%)	0.472
	No	73 (89.02%)	57 (85.07%)	

**Fig. 1:**
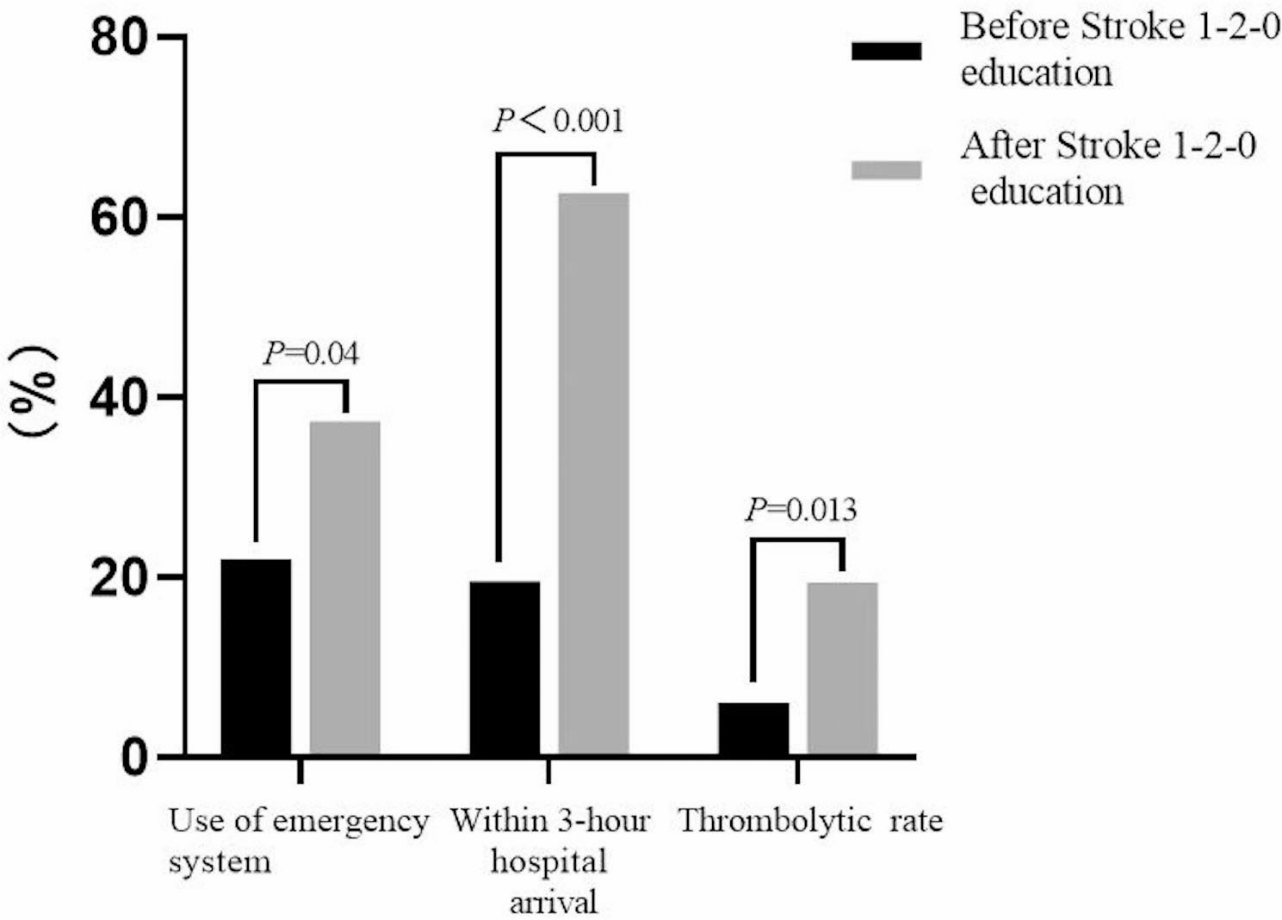
The effects of Stroke 1-2-0 education on stroke patients in the community

Figure 1 presents the prehospital data on stroke patients before and after the community education program. Regarding use of the emergency system, more patients with stroke after the program activated the 120 emergency system (21.95% vs. 37.31%; *P* = .04). The 3-hour arrival rate after the program was nearly 3 times higher than that before the program (62.69% vs. 19.51%; *P* < .001). In addition, thrombolysis treatment after stroke also increased substantially (after vs. before the program, 19.4% vs. 6.1%; *P* = .013). This indicates that the Stroke 1-2-0 community education program was effective.

## Discussion

Stroke is the predominant cause of mortality and disability among the adult population of China, and it is characterized by elevated rates of recurrence, morbidity, and incidence [13]. Early treatment is associated with a greater treatment efficacy and a reduced risk of complications in the context of acute ischemic stroke, which is treated within a brief time window [14, 15]. The latest guidelines for the early management of acute stroke suggest that thrombolytic therapy for ischemic stroke is most effective within 3 to 4.5 h of onset [16]. Prehospital and in-hospital delays are the main reasons why patients with acute ischemic stroke do not present within the narrow time window for thrombolytic therapy [17–20]. In particular, the prehospital delay caused by patients and the public is mainly reflected in the lack of awareness of stroke symptoms and the failure to make timely emergency calls [21]. In the past, stroke education for community residents was often carried out by specialist physicians entering the community or by offering education to hospitalized patients. However, this required the energy of the specialists, and many individuals who received the education soon forgot its content. The outcomes frequently yielded unsatisfactory results, ultimately leading to a loss of knowledge retention due to the absence of information repetition. Through our study, we found that the Stroke 1-2-0 prehospital education team composed of community general practitioners could significantly improve the knowledge of stroke among community residents, significantly shorten the prehospital delay after a stroke, improve the usage rate of the emergency system, and give more stroke patients the opportunity to undergo thrombolytic therapy.

Previous studies have utilized different stroke education models, such as television advertising, new media, and specialized education, to provide prehospital education for stroke. These studies have generally yielded positive outcomes, primarily in the short-term [22–24]. Large-scale stroke education through social media is often costly and requires the continuous consumption of sizable resources while not necessarily achieving sustained educational effects. In contrast, the cost of stroke education focused on the community environment is lower. The establishment of a team of neurologists from community general practitioners in conjunction with general hospitals can greatly reduce the cost of project support. It can also strengthen the relationships among physicians and neurologists, thus improving the effects of education. With the growth of general practice in China and the country’s rapid economic development in recent years, it has become critical to establish community general practitioner teams, which serve a critical role in maintaining the health of community members [25]. Our findings suggest that community general practitioners not only receive the stroke-related knowledge taught by specialists but can also apply it to the community residents’ stroke education, so that more people within the community can benefit.

With the aging of China’s population, the burden of stroke will continue to increase. Older people also often suffer from comorbidities, making the early detection and treatment of stroke in this age group especially important. That is, it is critical to improve the timely identification and treatment of stroke in older people. Early identification tools for stroke can help community residents correctly identify a stroke when it happens. The use of FAST identification rules for stroke education in foreign countries has significantly reduced the burden of disease due to stroke [26]. Previously, China attempted to employ a translated version of FAST for stroke education. However, the desired results were not achieved [8]. The current early thrombolysis rate of stroke in China is below 10%, which is significantly lower than the early stroke thrombolysis rate in developed countries in Europe and the United States [27]. To this end, Chinese physicians put forward the Stroke 1-2-0 stroke identification rule by integrating China’s emergency number 120 into the stroke identification tool, giving the tool distinct Chinese characteristics [9]. It is worth noting that the Stroke 1-2-0 educational campaign has made some progress in the field [28]. However, the current application of the Stroke 1-2-0 stroke identification tool in China is still insufficient, and more medical evidence is urgently needed to verify its value. Recently, our team published a population-based cross-sectional study, which suggests that adopting Stroke 1-2-0 can improve health outcomes and reduce clinical burdens for patients with stroke [29]. Moreover, Li et al. designed Stroke 1-2-0 health education cards and applied them to the education of patients before cerebral artery stenting. The results found that Stroke 1-2-0 can improve knowledge of patients and their families regarding stroke, making them more proactive about stroke treatment and reducing the mortality and morbidity rates of hospitalized patients [30]. However, Huang et al. surveyed 31 individuals from Guangdong, Hubei, Henan, Hebei, Hunan, and Liaoning through interviews and found that only about 50% of them knew about the three symptoms included in Stroke 1-2-0 [31]. Furthermore, a cross-sectional survey of community‐living older adults indicated that there is poor awareness of the stroke 1-2-0 educational tools [32]. These results show that the current Stroke 1-2-0 protocol in China still needs multifaceted publicity to improve the public’s awareness of it.

There are some strengths in our study. First off, this study is based on the customized Stroke 1-2-0 identification method tailored to China’s national conditions, specifically addressing the limitations of traditional FAST identification method in China. Second, this study provides a feasible method of providing stroke education through community general practitioners, making practical contributions to improving the stroke emergency response system. Last but not least, our study used before-and-after comparisons and cross-time data collection methods to provide a comprehensive assessment of the effects of educational interventions. However, our study also has some limitations that need to be considered. First, our study sample was limited to community general practitioners and residents in Minhang District of Shanghai, which may limit the general applicability of our conclusions. Second, because this is a prospective intervention study, we cannot rule out that factors other than educational interventions may have had an impact on the results. In addition, our findings rely primarily on self-reported data, which can be affected by recall bias. To address these constraints, future research should investigate employing the design of randomized controlled trials to increase sample size and cover more locations and populations to improve the generalizability of the results.

## Conclusion

We found that a Stroke 1-2-0 prehospital education team consisting of community general practitioners could significantly improve stroke knowledge, shorten prehospital delays after a stroke, and increase emergency system usage. As a result, more stroke patients were able to receive thrombolytic therapy.

## Data Availability

The data that support the findings of this study are available from the corresponding author upon reasonable request.
